# Hydrogen Sulfide Mediating both Excitatory and Inhibitory Effects in a Rat Model of Meningeal Nociception and Headache Generation

**DOI:** 10.3389/fneur.2017.00336

**Published:** 2017-07-14

**Authors:** Christiane Teicher, Roberto De Col, Karl Messlinger

**Affiliations:** ^1^Institute of Physiology and Pathophysiology, University of Erlangen-Nürnberg, Erlangen, Germany

**Keywords:** nitroxyl, hydrogen sulfide, nitric oxide, trigeminal nucleus caudalis, meningeal nociception, extracellular recording, migraine, headache

## Abstract

**Background/purpose:**

Hydrogen sulfide (H_2_S) is a neuromodulator acting through nitroxyl (HNO) when it reacts with nitric oxide (NO). HNO activates transient receptor potential channels of the ankyrin type 1 (TRPA1) causing release of calcitonin gene-related peptide from primary afferents. Activation of meningeal nociceptors projecting to the human spinal trigeminal nucleus (STN) may lead to headaches. In a rat model of meningeal nociception, the activity of spinal trigeminal neurons was used as read-out for the interaction between H_2_S and NO.

**Methods:**

In anesthetized rats extracellular recordings from single neurons in the STN were made. Sodium sulfide (Na_2_S) producing H_2_S in the tissue and the NO donor diethylamine-NONOate (DEA-NONOate) were infused intravenously. H_2_S was also locally applied onto the exposed cranial dura mater or the medulla. Endogenous production of H_2_S was inhibited by oxamic acid, and NO production was inhibited by nitro-l-arginine methyl ester hydrochloride (l-NAME) to manipulate endogenous HNO formation.

**Key results:**

Systemic administration of Na_2_S was followed either by increased ongoing activity (in 73%) or decreased activity (in 27% of units). Topical application of Na_2_S onto the cranial dura mater caused a short-lasting activation followed by a long-lasting decrease in activity in the majority of units (70%). Systemic administration of DEA-NONOate increased neuronal activity, subsequent infusion of Na_2_S added to this effect, whereas DEA-NONOate did not augment the activity after Na_2_S. The stimulating effect of DEA-NONOate was inhibited by oxamic acid in 75% of units, and l-NAME following Na_2_S administration returned the activity to baseline.

**Conclusion:**

Individual spinal trigeminal neurons may be activated or (less frequently) inhibited by the TRPA1 agonist HNO, presumably formed by H_2_S and NO in the STN, whereby endogenous H_2_S production seems to be rate-limiting. Activation of meningeal afferents by HNO may induce decreased spinal trigeminal activity, consistent with the elevation of the electrical threshold caused by TRPA1 activation in afferent fibers. Thus, the effects of H_2_S–NO–TRPA1 signaling depend on the site of action and the type of central neurons. The role of H_2_S–NO–TRPA1 in headache generation seems to be ambiguous.

## Introduction

Hydrogen sulfide (H_2_S) is a toxic gas released from natural and artificial sources (1), but at very low concentrations it acts within the body as a signal molecule in the cardiovascular and the central nervous system (2, 3). Endogenous H_2_S is cleaved from cysteine by pyridoxal-5′-phosphate-dependent enzymes, particularly cystathionine β-synthase (CBS) and cystathionine γ-lyase (4–8). In addition to its cardiovascular and central functions, H_2_S exerts various biological effects like insulin secretion from rat pancreatic B-cells (9), facilitation of carotid sinus baroreceptor activity (10), and relaxation of human airway smooth muscle cells (11) through activation of K_ATP_ channels. H_2_S may inhibit Ca^2+^-activated K^+^ channels and the Na^+^-K^+^-ATPase, thus contributing directly to depolarization of pulmonary epithelial cells (12). An overproduction of H_2_S may be implicated in severe diseases like sepsis and colorectal cancer, where it seems to be a cell protective factor, while in other diseases H_2_S levels are reduced (13). H_2_S-mediated signaling pathways are not only mediated by the intact compound but also by its oxidized forms, polysulfides.

Because of its neuromodulating effects in the nervous system, H_2_S has been regarded as the third “gasotransmitter” besides nitric oxide (NO) and carbon monoxide (14). H_2_S and NO react preferably to form nitroxyl (HNO), the one-electron-reduced sibling of NO, which has been shown to be a strong activator of transient receptor potential (TRP) channels of the ankyrin type 1 (TRPA1) (15). TRPA1 channels are expressed in a subset of nociceptive primary spinal and trigeminal afferents, mainly co-existent with TRP channels of the vanilloid type 1 (TRPV1) (16–18). Opening of TRPA1 and TRPV1 receptor channels in primary afferent neurons at normal membrane potentials allows inward currents of Na^+^ and Ca^2+^, which depolarize the cell membrane and lead to the exocytosis of neuropeptides like substance P and calcitonin gene-related peptide (CGRP) in neurons storing these peptides (19, 20). This signaling cascade has recently been demonstrated in a variety of *in vivo* and *in vitro* experiments in our laboratory to cause CGRP release from trigeminal afferent fibers in the rat dura mater followed by increased meningeal blood flow (21).

Similar signaling mechanisms as in peripheral tissues have frequently been found in the spinal and medullary dorsal horn. Indeed, there is histological evidence for H_2_S–NO–CGRP signaling in the spinal trigeminal nucleus (STN) (21), where the central trigeminal terminals project to second order neurons. We hypothesized therefore that the gasotransmitters H_2_S and NO are also involved in modulating the activity of these central neurons. The second order neurons in the STN receiving primary afferent input from the cranial meninges (22, 23) may have a pivot function in central nociceptive processing that causes the sensation of headaches. A putative role of TRPA1 receptor channels in the pathogenesis of headaches has particularly been discussed after recognizing that TRPA1 agonists like umbellulone, a product of the “headache tree,” may initiate headache and migraine attacks (24). Thus, assuming that the endogenous production of H_2_S and NO forming HNO (25, 26) contributes to the control of neuronal activity by activating TRPA1 receptors and CGRP release in the STN (15), we tried to manipulate this signaling cascade by application of an H_2_S and an NO donor and inhibiting endogenous H_2_S or NO production using the spinal trigeminal activity as a read-out in an established animal model of meningeal nociception. The results indicate that individual spinal trigeminal neurons may be activated or inhibited by H_2_S–NO–CGRP signaling, possibly depending on the site of action and the type of central neurons involved.

## Materials and Methods

### Anesthesia and General Preparation

The experiments were performed on adult male Wistar rats with body weights of 250–350 g, bred and housed in the animal facility of our Institute. All experiments were done in accordance with the ethical guidelines of the International Association for the Study of Pain and in compliance with the guidelines for the welfare of experimental animals of the Federal Republic of Germany and the European Commission (Directive 2010/63/EU). The experimental protocol was reviewed by an ethics committee and approved by the District Government of Unterfranken.

For anesthesia, the animals were placed into a closed box, which was filled with an air–oxygen-mixture and isoflurane (Forane^®^ Vapor 19.3, Drägewerk AG, Lübeck, Germany) at increasing concentrations up to 4%; then isoflurane at 2.5% was applied through a mask for surgical procedures. The right femoral artery was catheterized to monitor arterial blood pressure, which ranged between 70 and 120 mmHg. The catheter system contained heparin sodium 5000 (Ratiopharm GmbH, Ulm, Germany) in a 1:5,000 solution with sodium chloride 0.9% (B. Braun Melsungen AG, Melsungen, Germany). Furthermore, the right femoral vein was cannulated to allow infusion of solutions and drugs. To guarantee safe machine-assisted ventilation with oxygen-enriched room air over several hours, the rats were intubated with an intravenous cannula (Vasuflo^®^-T G14, Dispomed Witt, Germany). Atropine sulfate (B. Braun Melsungen AG, Melsungen, Germany, 0.5 mg/ml 1:10 with sodium chloride 0.9%) was injected subcutaneously to prevent salivation and muscle spasms.

Physiological monitoring of vital parameters (mean blood pressure, expiratory CO_2_ levels, respiratory rate, and body temperature) was ensured throughout the experiment. To maintain a constant body temperature of 37.9–38.2°C, each rat was placed on a heating plate connected to a feedback-controlled homeothermic system (TKM 0902, FMI GmbH, Seeheim Ober-Beerbach, Germany). During the experiment, an isoflurane concentration of 2% was adequate to maintain a constant depth of anesthesia, deactivated motor reflexes to noxious pinch stimuli of the hind paw and a constant blood pressure. The end-expiratory CO_2_ was monitored by a sensor (HeyerArtema MM 200, Bad Ems, Germany) and kept at 2.5–3.0% to suppress any spontaneous breathing. In case of a ventilation resistance exceeding 10 mmHg, saliva was removed with a blunt plastic cannula through the tracheal tube.

### Head Surgery

The rat’s head was fixed in a stereotactic frame and held by ear bars and a snout clamp. To avoid activation of nociceptors in the auditory canal, local anesthetic ointment containing lidocaine (Posterisan^®^ akut, Dr. Kade Pharma, Konstanz, Germany) was used. The eyes were covered with dexpanthenol ointment (Bepanthen^®^, Bayer Vital GmbH, Leverkusen, Germany) to prevent dehydration of the cornea. A median incision was made along the midline of the scalp and the skin of the neck. A cranial window of about 10 mm × 7 mm was carefully cut into the right parietal bone using a dental drill (KaVo EWL, max. 25,000/min, KaVo Dental GmbH, Biberach, Germany) under saline rinsing. The exposed dura was covered with isotonic saline throughout the experiment. Then, the medulla oblongata containing the caudal part of the STN was exposed. The neck muscles and the dorsal atlanto-occipital ligament with the underlying dura mater were incised and pulled apart to expose the cisterna cerebellomedullaris (c. magna). In some experiments, parts of the atlas vertebra were excised to gain access to the upper cervical segments.

### Stimulation and Data Recording

**Figure 1:**
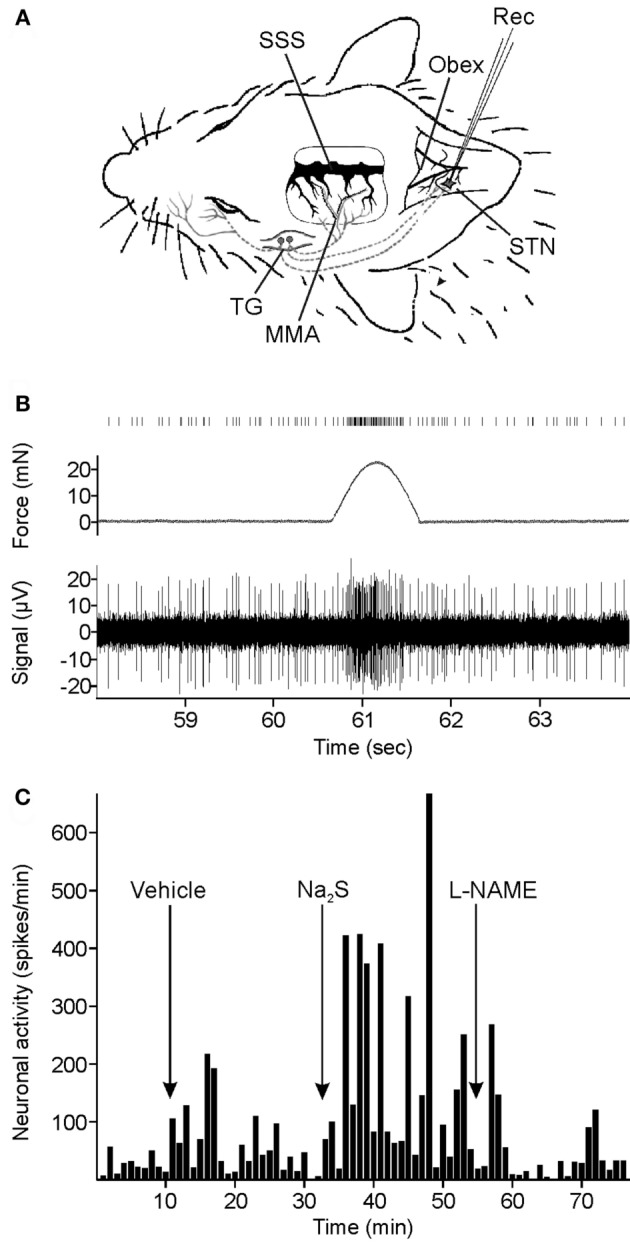
Experimental setup and data recording. **(A)** Extracellular recording (Rec) of neuronal activity in the spinal trigeminal nucleus (STN). Meningeal afferents were stimulated by repetitive mechanical stimuli applied to the exposed parietal dura mater in a cranial window around the middle meningeal arteria (MMA). SSS, superior sagittal sinus; TG, trigeminal ganglion. **(B)** Original recording of neuronal activity. Repetitive mechanical stimuli elicit clusters of action potentials in addition to the ongoing activity. The counted spikes in the offline analysis are presented on top. **(C)** Spike histogram of ongoing neuronal activity and changes after systemic administration of vehicle, sodium sulfide (Na_2_S), and nitro-l-arginine methyl ester hydrochloride (l-NAME) in a typical experiment.

For extracellular recording from single neurons in the STN, custom-made carbon fiber glass microelectrodes with an impedance of about 1 MΩ were slowly advanced at 2.5 µm steps through the ipsilateral medullary brainstem driven by a nanostepper (SMS87 TC Elektronik, Würzburg, Germany) (Figure 1A). Neurons in the subnucleus caudalis of the STN with meningeal nociceptive input were detected by their spiking evoked by touching the parietal dura with von Frey filaments (2.9–11.8 Nm). Besides their responses to mechanical stimulation of the dura, the recorded neurons matched the criteria of being spontaneously active with a minimal steady frequency of one per second and action potentials with amplitudes of at least 20 mV. Prior to recording, a muscle relaxant (gallamine triethiodide, Sigma G 8134, Sigma-Aldrich Chemie GmbH, Germany, 50 mg/ml sodium chloride 0.9%) was i.v. infused (initially 0.3 ml, then 0.2 ml every 2 h) to avoid muscular contractions to electrical stimuli.

Convergent input from extracranial tissues was mapped by probing the ipsilateral cornea, temporal muscle, periosteum, and facial skin (V1, 2, 3) with von Frey filaments and a fine blunt glass rod. A custom-made mechanostimulator was placed on the meningeal receptive field to deliver impulses of 1-s duration every minute with an optimized force (range 15–25 mN, about three times the von Frey filaments threshold) eliciting a cluster of 5–20 action potentials. The data were recorded with the computer software Spike2 6.08 (Cambridge Electronic Design, Cambridge, UK) and saved for later offline analysis (Figure 1B). After finishing the recordings, electrical single shock stimuli were applied with bipolar electrodes to the exposed parietal dura to determine the electrical threshold just activating the neuron. In addition, responses to topically applied capsaicin (10^−6^ M) were assessed.

At the end of each experiment, the coordinates of the electrode in relation to the obex as well as the depth of the recording site were measured. The experiments were terminated by i.v. injection of a lethal dose of 6% thiopental (Trapanal^®^, Nycomed Deutschland GmbH, Konstanz, Germany).

### Chemicals

A stock solution of 100 mM sodium sulfide (Na_2_S) (Department of Chemistry and Pharmacy, FAU Erlangen) was diluted to 300 µM with synthetic interstitial fluid (SIF) for topical application or to 900 µM with saline for intravenous infusion. The CGRP receptor antagonist Olcegepant (BIBN4096BS, Boehringer Ingelheim Pharma KG, Germany) was dissolved in acidic pH at 1 mg/ml and then titrated to neutral values. The nitric oxide synthase (NOS) inhibitor nitro-l-arginine methyl ester hydrochloride (l-NAME) (Sigma-Aldrich Chemie GmbH, Taufkirchen, Germany) was dissolved in saline for intravenous infusion at a dose of 10 mg/kg. The NO donor diethylamine-NONOate (DEA-NONOate) (Merck Millipore, Schwalbach, Germany) was dissolved in 10 mM NaOH and diluted in saline immediately before intravenous infusion at a dose of 2.5 µg/kg. An inhibitor of H_2_S synthesis, oxamic acid (aminooxyacetic acid) (Sigma-Aldrich Chemie GmbH, Taufkirchen, Germany), was dissolved in saline for intravenous infusion (3 mg/kg).

### Experimental Protocols

Each experiment started with a control stimulation period (baseline recording) followed by intravenous infusion of saline or topical application of SIF as a vehicle. After the test stimulation period, a post-control period was added. Each recording period following application of any substance lasted 20 min, which is long enough to assure changes in neuronal activity caused by experimental manipulations but allows also a sequence of several recording periods within a reasonable time.

In the first group of 19 animals, Na_2_S was topically (50 µl, 300 µM) applied to the exposed dura and/or to the spinal medulla and/or slowly injected intravenously (i.v., 0.3 ml, 900 µM) over 2 min in arbitrary order. In nine experiments Na_2_S was administered to the dura and/or the medulla and injected i.v., in eight experiments either to the dura or the medulla or i.v. Subsequently, l-NAME (0.5 ml, 10 mg/kg) was added intravenously (Figure 1C). Finally, in five experiments Olcegepant was i.v. injected 20 min after l-NAME at a dose of 1 mg/kg to check if activated CGRP receptors were still present contributing to the ongoing activity.

In the second test group, 14 neurons (in 14 animals) were infused by DEA-NONOate (0.2 ml, 2.5 µg/kg) followed by oxamic acid (0.2 ml, 3 mg/kg) in eight cases. The infusion was held at a low constant rate of 1.2 ml/h to avoid changes in systemic blood pressure possibly induced by these vasoactive substances. The activity was then recorded for other 30 min.

In the third test group of six animals, the effect of i.v. administration of Na_2_S (0.25 ml) before and after DEA-NONOate (0.2 ml) was tested. Each substance was infused over a period of 5–6 min (2.4 ml/h), and the recording was continued for 20 min.

### Data Analysis

The neuronal activity of recorded neurons was analyzed with Spike2 6.08 (Cambridge Electronic Design, Cambridge, UK) after it had been cleaned from artifacts using the touch-evoked responses as templates. The number of spikes was counted and listed at intervals of minutes (partly also seconds) with Excel software 2013 (Microsoft Corporation, USA) to determine mean values of activity and SEM (mean ± SEM). For calculating the ongoing activity, the mechanically evoked activity was subtracted. The mechanically evoked activity is the activity during the 1-s mechanostimulation interval diminished by the mean ongoing activity within 1 s before and 1 s after stimulation. For generating diagrams, the ongoing and the mechanically evoked activity was normalized to the mean baseline activity recorded during vehicle.

For statistical analysis Statistica 7.0 software (StatSoft, Tulsa, OK, USA) was used. The neuronal activity of each individual unit counted at 1-min intervals was analyzed with one-way ANOVA followed by Fisher’s least significance difference (LSD) *post hoc*-test to compare the variance in activity before and after drug administration. The activity of units within each particular group was then evaluated by ANOVA with repeated measurements, also followed by Fisher’s LSD *post hoc*-test. Furthermore, the Wilcoxon matched pairs test (for *n* < 10) or the Student’s *t*-test (*n* ≥ 10) was performed for comparing the neuronal activity before and after administration of drugs. Differences were regarded significant at *p* < 0.05.

The diagrams were created with Origin 7 software (OriginLab Corporation, USA) and edited by CorelDRAW Graphics Suite X8 software (Corel Corporation, Canada).

## Results

### General Properties of Neurons

Recordings were made from 32 neurons in the STN of 32 rats. The neurons were characterized by their afferent input from the exposed ipsilateral parietal dura mater; the receptive fields were mostly located close to the middle meningeal artery. Additionally, convergent input from the ophthalmic, maxillary, and mandibular areas of the facial fur, the temporal or neck muscles, and the periosteum around the cranial window was determined. All neurons but one responded to touch or light pressure applied to facial receptive fields in addition to their meningeal input and were therefore classified as wide dynamic range neurons.

The recording sites were located 1.1/1.8/3.0 mm (min/mean/max) caudal to the obex, 0.6/1.2/1.9 mm ipsilateral from the midline and at depths of 98/748/1,327 μm below the dorsal surface of the medulla. The ongoing activity of the recorded units during the control period varied between 97 and 2,038 (mean 497 ± 91) spikes per minute. Nearly all units located in superficial layers of the trigeminal nucleus (≤500 µm) had low spontaneous activity (<400/min), but in the whole sample the depth of recording sites and the unit’s spontaneous activity showed no significant correlation.

The mechanical threshold determined by graded von Frey filaments ranged between 0.98 and 6.9 mN (mean 4.1 ± 0.3 mN). The electrical threshold identified by electrical square pulses (duration 1 ms) applied to the dural receptive field at the end of the experiments ranged from 0.02 to 8 mA (mean 2.33 ± 0.64 mA). The nerve fibers responded with latencies between 5 and 25 ms (mean 16 ± 1.9 ms) to single electrical pulses slightly above threshold. Assuming an average distance of 25 mm from the stimulation area on the dura to the recording site in the brainstem, the afferent fibers activating the units could be classified as Aδ-fibers (>2 m/s) or C-fibers (<2 m/s) at a proportion of about 40/60%. Twelve units were tested with capsaicin topically administered to the exposed dura mater as a chemical stimulus for nociceptors. All tested units responded immediately with clusters of five to twenty action potentials.

### Neuronal Responses to Na_2_S/H_2_S

Hydrogen sulfide has been defined as a neuromodulator in nociceptive processing (27). The first series of experiments was made to examine if systemic or local application of H_2_S modulates the activity of spinal trigeminal neurons processing meningeal afferent information. We hypothesized that H_2_S binds to endogenous NO to form HNO that may activate TRPA1 receptor channels of primary meningeal afferents resulting in an increased spinal trigeminal activity.

#### Vehicle Control

In seven units the spontaneous baseline activity was recorded for 20 min, and the variance in activity in each minute was compared to the variance in activity within 20 min after i.v. injection of saline as a vehicle control. ANOVA analysis at 1-min intervals yielded four units without changes, two units with increased activity after saline, and one unit with decreased activity. In another sample of 10 units, the activity was recorded 20 min before and after topical application of vehicle (saline) to the exposed dura mater. In eight units, there was no change, and in two units the activity decreased after vehicle. In the two samples of units, there was no significant change in activity over the control time.

#### Systemic Administration of Na_2_S

**Figure 2:**
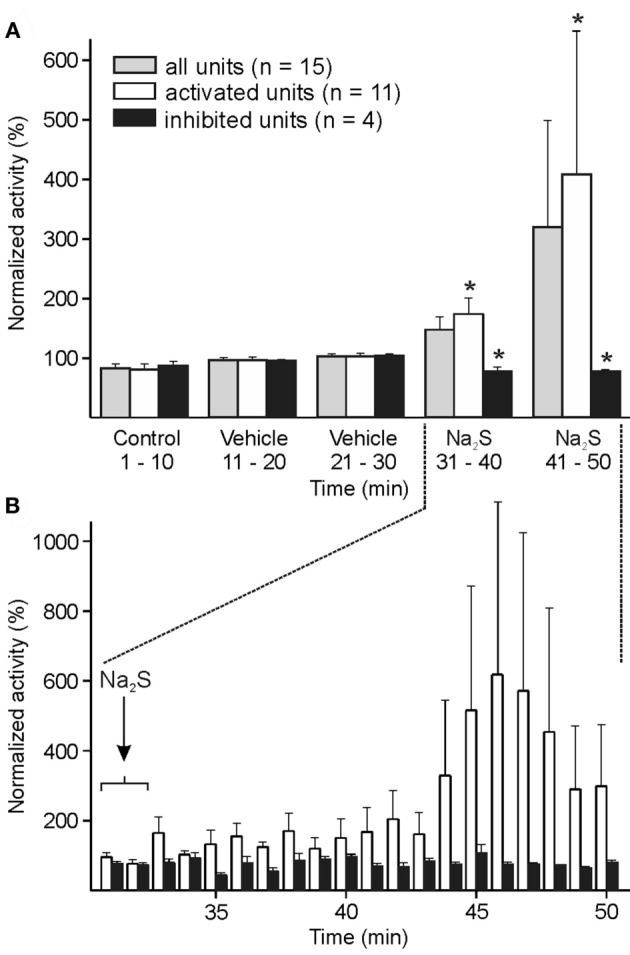
Activity of units displayed at 10-min **(A)** or 1-min intervals **(B)** normalized to the vehicle interval before systemic administration of sodium sulfide (Na_2_S). **(A)** The whole sample of 15 units showed no significant change in activity after Na_2_S. When each single unit was analyzed separately with one-way ANOVA at minute intervals, the neuronal activity was significantly increased in 11 units and decreased in 4 units after Na_2_S infusion. In these two groups of activated/inactivated units the activity after Na_2_S was significantly different to the baseline/vehicle (*) intervals (ANOVA with repeated measures). **(B)** Higher resolution showing increased activity after Na_2_S administration in the activated group of units.

In 15 units, ongoing and mechanically evoked activity was recorded 20 min before and after i.v. infusion of Na_2_S. Although an increase in ongoing activity after Na_2_S infusion was apparent, it was statistically not significant in the whole sample of experiments. The analysis of each single unit with one-way ANOVA at 1-min intervals revealed 11 units with higher activity after Na_2_S, while 4 units showed a significant decrease in activity after Na_2_S compared to baseline/vehicle. In the group of the 11 neurons, the activity analyzed within 10-min intervals was significantly higher after Na_2_S compared with the intervals after baseline and vehicle [ANOVA with repeated measurements, *F*(4, 40) = 5.651, *p* < 0.01; followed by LSD test, *p* < 0.01–0.05] (Figures 2A,B).

The mechanically evoked activity did not show any significant change in activity, regardless if all units or the units with increased spontaneous activity after Na_2_S administration were analyzed.

#### Dural Administration of Na_2_S

**Figure 3:**
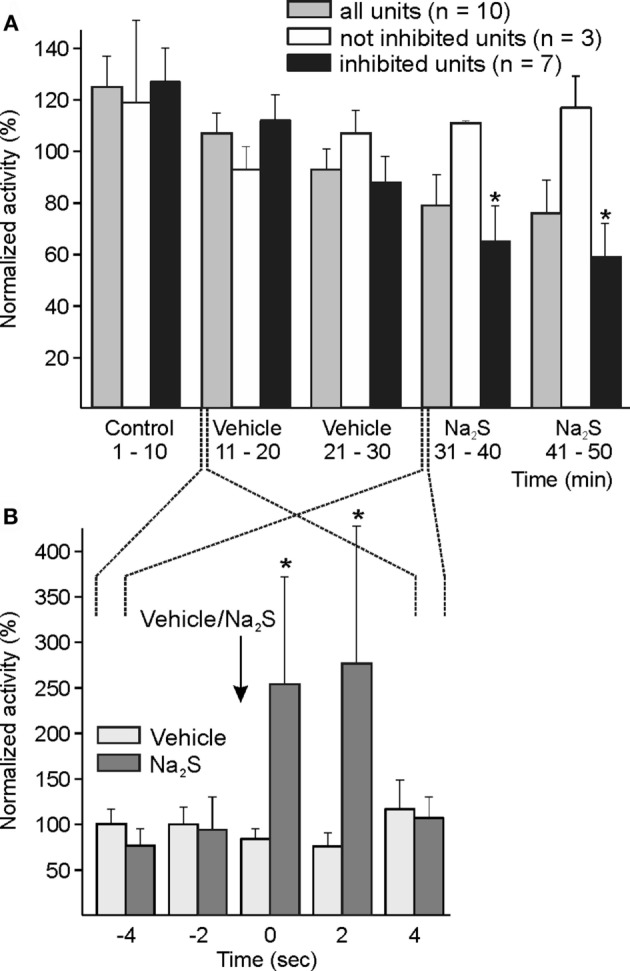
Activity of units displayed at 10-min **(A)** and 2-s intervals **(B)** normalized to the respective vehicle intervals, and changes after dural administration of sodium sulfide (Na_2_S). **(A)** The whole sample of 10 units showed a tendency of decreased activity without significant change following local application of Na_2_S onto the dura mater. The analysis of each single unit (one-way ANOVA at minute intervals) resulted in a significant decrease in neuronal activity in seven units. In this group, the decrease in activity was significant compared to baseline/vehicle (*) intervals (ANOVA with repeated measures). Three units were not inhibited by topical Na_2_S. **(B)** Higher resolution at 2-s intervals showed a short-lasting significant increase in activity between one interval before and the first two intervals after Na_2_S application (*) but not after vehicle.

Ten units were recorded during topical application of Na_2_S onto the dura mater. The activity of the whole cohort analyzed at intervals of 1 and 10 min tended to decrease without statistically significant variation. The analysis of individual experiments by one-way ANOVA showed that the ongoing activity in 7 of 10 units decreased after Na_2_S, in 2 units there was no change, and in one the activity was increasing. In the group of seven units, the decrease in neuronal activity was significant compared to the baseline/vehicle activity analyzed at intervals of 10 min [ANOVA with repeated measurements, *F*(5, 30) = 2.632, *p* < 0.05; LSD test, *p* < 0.05] (Figure 3A).

The mechanically evoked activity did not significantly change after Na_2_S application.

In primary meningeal afferent recordings *in vitro*, we observed a short-lasting transient excitation after topical application of the HNO donor Angeli’s salt (unpublished experiments). Because in some of the present experiments a short-lasting activation immediately following Na_2_S application onto the dura mater was obvious, we evaluated the activity of the whole sample at intervals of 2 s around the application, resulting in a significant increase in activity for 4 s after Na_2_S application [ANOVA with repeated measurements, *F*(3, 24) = 3.339, *p* < 0.05; LSD test *p* < 0.05] (Figure 3B). The application of vehicle was not followed by any comparable change in activity.

#### Medullary Administration of Na_2_S

**Figure 4:**
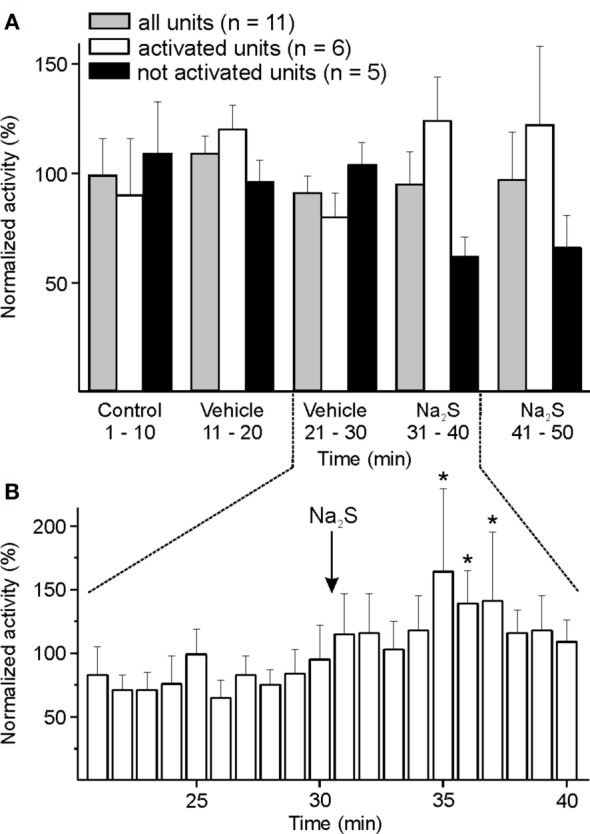
Activity of units displayed at 10-min **(A)** or 1-min intervals **(B)** normalized to the respective vehicle intervals, and changes after medullary administration of sodium sulfide (Na_2_S). **(A)** The whole sample of 11 units showed no significant change in activity after local administration of Na_2_S onto the spinal medulla. When each single unit was evaluated separately (one-way ANOVA at minute intervals), the neuronal activity of six units was increasing after Na_2_S but not after vehicle administration. **(B)** Higher resolution revealed increased activity (*) in the group of activated units in minutes 5–7 after starting Na_2_S administration compared to the 10-min interval before Na_2_S infusion (ANOVA with repeated measures).

Responses to topical application of Na_2_S/H_2_S onto the spinal medulla were examined in 11 units. The whole group did not show any significant change in activity. Analyzing individual units, an increased activity was visible in six units. The activity of these six units increased significantly between minute 5 and 7 after administration of Na_2_S compared to the 10 min before drug application [ANOVA with repeated measurements, *F*(19, 95) = 1.935, *p* < 0.05; followed by LSD test, *p* < 0.05] (Figures 4A,B). There was no change in activity following vehicle administration.

The mechanically evoked activity did not significantly change after Na_2_S application.

### Neuronal Interaction between Na_2_S and DEA-NONOate

Hydrogen sulfide interacting with NO may be more effective than H_2_S alone, which has recently been concluded from measurements of CGRP release from medullary slices (28). Therefore, in a variety of experiments we combined Na_2_S and DEA-NONOate and inhibited the endogenous production of H_2_S by oxamic acid or the NO production by l-NAME, which has previously been shown to lower the activity of spinal trigeminal neurons with meningeal afferent input (29). The CGRP receptor inhibitor, BIBN4096BS (Olcegepant), was eventually applied.

#### Vehicle Control

In 21 units, the spontaneous activity was recorded for 20 min and the variance in activity between the minutes compared to the variance in activity within 20 min after infusion of vehicle (saline). In five units, there was no change, in eight units, the activity increased, and in eight others it decreased. The whole group showed no significant change in activity.

#### Systemic Administration of DEA-NONOate

**Figure 5:**
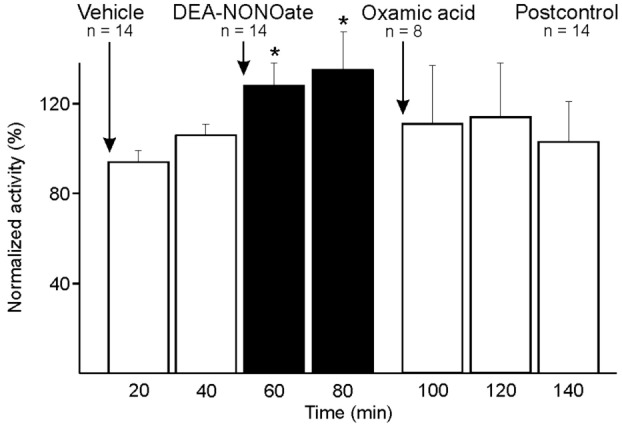
Activity of units displayed at 20-min intervals normalized to the activity during vehicle, and changes after systemic administration of diethylamine-NONOate (DEA-NONOate) and oxamic acid. In the sample of 14 units the activity increased after DEA-NONOate (*) compared to vehicle (two-way ANOVA with repeated measures). After subsequent administration of oxamic acid in eight units the activity returned to baseline.

The NO donor DEA-NONOate was systemically administered in 14 experiments. The neuronal activity was recorded over 40 min (10-min infusion period and subsequently 30 min) and evaluated at 20-min intervals. During infusion of DEA-NONOate an increased activity was apparent followed by a second phase of increased neuronal activity over the next 30 min [two-way ANOVA with repeated measurements, DEA-NONOate compared to vehicle, *F*(1, 13) = 21.646, *p* < 0.001; LSD test, *p* < 0.001–0.01] (Figure 5).

The mechanically evoked activity did not significantly change after DEA-NONOate administration.

#### Systemic Administration of Na_2_S Combined with DEA-NONOate

**Figure 6:**
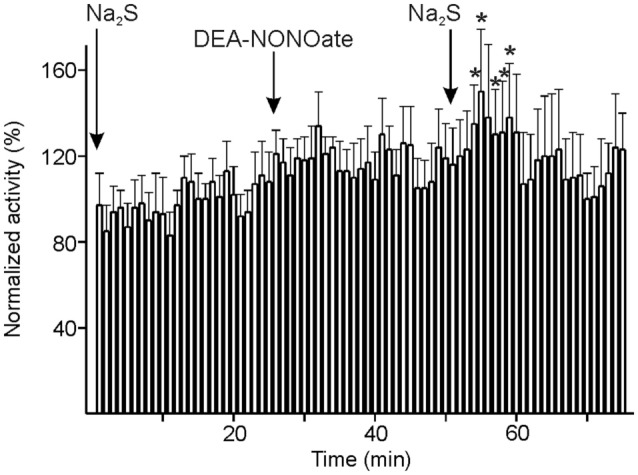
Activity of units displayed at 1-min intervals normalized to the vehicle intervals and changes after consecutive systemic administration of sodium sulfide (Na_2_S), diethylamine-NONOate (DEA-NONOate) and a second Na_2_S stimulus. The six units showed no significant change in activity after the first Na_2_S and the DEA-NONOate infusion but a significant increase (*) in minutes 4–9 after starting the second Na_2_S infusion compared to the 10-min intervals before the respective drug application (ANOVA with repeated measures).

In six units, Na_2_S and DEA-NONOate were administered intravenously at intervals of 20 min followed by a second Na_2_S administration. The neuronal activity tended to increase after the first Na_2_S infusion and after DEA-NONOate; after the second Na_2_S infusion there was a rapid increase in activity, particularly during minute 4–9 after start of the infusion [ANOVA with repeated measurements, *F*(24, 96) = 1.6813, *p* < 0.05; LSD test, *p* < 0.05] (Figure 6). Looking at the six individual units, there was a significant change in activity between Na_2_S before and after DEA-NONOate in each case: it was increasing in four (one-way ANOVA, *p* < 0.01; LSD test, *p* < 0.001) and decreasing in two units (one-way ANOVA, *p* < 0.01; LSD test, *p* < 0.001).

There was no significant change in the mechanically evoked activity.

### Inhibition of Endogenous H_2_S and NO Synthesis

#### Systemic Administration of Oxamic Acid following DEA-NONOate

In eight experiments, the administered DEA-NONOate was followed by oxamic acid inhibiting endogenous H_2_S synthesis. Comparing 10 min before and after application of oxamic acid, the Wilcoxon matched pairs test detected a just significant decrease in activity (*p* < 0.05) (Figure 5), which was due to the majority of six units, while in two units the activity increased further after oxamic acid (one-way ANOVA of individual units, *p* < 0.001).

The mechanically evoked activity did not show significant changes.

#### l-NAME following Systemic Administration of Na_2_S

**Figure 7:**
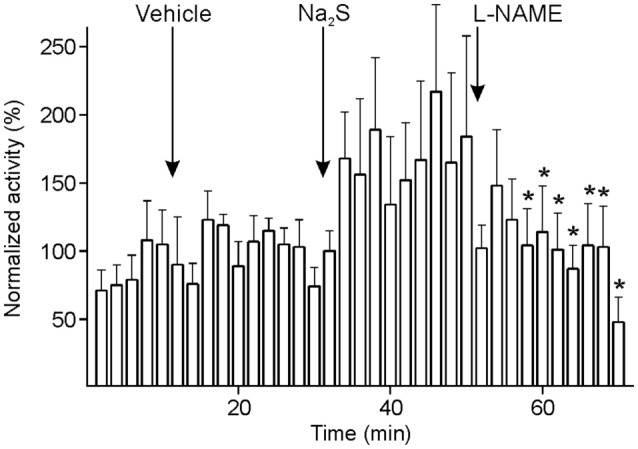
Activity of units displayed at 2-min intervals normalized to the activity during vehicle, and changes after systemic administration of sodium sulfide (Na_2_S) and nitro-l-arginine methyl ester hydrochloride (l-NAME). The sample of seven units returned to baseline after l-NAME with lower activity (*) compared to the activity after Na_2_S (ANOVA with repeated measures).

In seven experiments, the NO synthase inhibitor, l-NAME, was i.v. applied 20 min after Na_2_S administration to figure out if endogenous NO production is required for the long-lasting increase in activity following Na_2_S administration in the majority of units. Since the activity seemed to fluctuate within short periods after the administration of l-NAME, it was analyzed with higher resolution at 2-min intervals. The activity tended to increase after Na_2_S administration. From 6 min after l-NAME it decreased significantly compared to minute 2–14 after Na_2_S application and was finally below the level of baseline and vehicle [ANOVA with repeated measurements, *F*(69, 414) = 1.382, *p* < 0.05; LSD test, *p* < 0.05] (Figure 7). Finally, the CGRP receptor inhibitor BIBN4096BS (olcegepant) added in five units did not lead to a further decrease in activity.

An increase in blood pressure between 10 and 50 mmHg (mean 28 mmHg) occurred 60–290 s after l-NAME administration.

The mechanically evoked activity did not significantly change after l-NAME administration.

## Discussion

### Neuronal Responses to Na_2_S/H_2_S

Spinal trigeminal neurons processing nociceptive information from the dura mater encephali seem to respond differentially to Na_2_S/H_2_S. Systemic application of Na_2_S caused activation in the majority of units (73%), whereas in a minor proportion the activity slowly decreased within 10–20 min after infusion of Na_2_S. The mechanically evoked activity remained unchanged. After topical administration of Na_2_S onto the spinal medulla these two different groups were also visible, though with minor significance. In contrast, topical administration of Na_2_S onto the cranial dura mater caused a very short-lasting activation followed by a slow and long-lasting decrease in activity in most of the units. These different behaviors were not correlated with any other criteria characterizing the units like basal spontaneous activity or convergent afferent input from facial areas.

#### Systemic Administration of Na_2_S

The seeming discrepancy of both activated and inhibited units warrants a discussion about the neuronal effects of H_2_S, which may vary depending on several factors. H_2_S is known as a potent activator of TRPA1 receptor channels (30) subserving pro-nociceptive actions of H_2_S in somatic tissues (27). TRPA1 activation is involved in sensory transduction in nociceptors, particularly during inflammation and oxidative stress (31). TRPA1 has been found functionally expressed primarily in the isolectin B_4_-positive, CGRP-negative subpopulation of small lumbar DRG neurons (32). Therefore, TRPA1 activated by HNO may initiate different signaling pathways resulting in the peripheral and central release of CGRP in a smaller proportion of primary afferents.

Besides its activating effect on TRPA1 receptor channels, H_2_S may be involved in multiple signaling pathways in the peripheral and central nervous system by targeting ion channels like T-type and L-type calcium channels, ATP-sensitive K^+^ (K_ATP_) channels and *N*-methyl-d-aspartate channels, which modulate nociceptive processes [reviewed in Ref. (33)]. H_2_S may also activate Ca^2+^-dependent K^+^ channels (BKCa), the delayed rectifier K^+^ current (I_K_) as well as one type of sodium channel (Na_v_1.5); and finally, TRPV1 and chloride channels are also suggested to be modulated by H_2_S (34). Due to its multiple actions, it is not surprising that H_2_S has been reported to act ambiguously, being beneficial or harmful depending on its concentration and cellular location (35). H_2_S at micromolar concentrations has cytoprotective and antioxidant effects, whereas H_2_S exposure in the millimolar range potentiates redox stress and is cytotoxic [reviewed in Ref. (34)]. The effective concentration of H_2_S in the organisms depends on several factors like pH, temperature, and oxygen, which may lead to opposite effects. In alkaline solutions, sulfide exists predominantly as highly reactive HS^−^ anion, and low temperatures lead to a higher percentage of H_2_S gas (36). H_2_S production is particularly increased in hypoxic conditions (37). Also, systemic cardiovascular effects of H_2_S may interact differentially with the neuronal responses. Infusions of the H_2_S donors, Na_2_S and NaHS, in rats decreased systemic arterial pressure and heart rate dose-dependently, which could not sufficiently be explained by distinct peripheral or central signaling mechanisms (38).

It is likely that the cardiovascular effects as well as a major part of H_2_S effects in the central nervous system including the spinal trigeminal system is attributed to the synthesis of HNO/NO^−^ in the presence of NO and H_2_S, leading to CGRP release from sensory neurons through TRPA1 activation (15). Thus, it is tempting to assume that the individual endogenous production of NO close to the recorded neuron may be another factor influencing H_2_S effects. Moreover, it is likely that not all spinal neurons are controlled by NO, and finally, H_2_S itself or the generated HNO might have an activating effect on inhibitory neurons, which could inverse the activation and eventually lead to an inhibition of the recorded unit. We propose this hypothesis based on preliminary findings of our group (unpublished): Patch clamp recordings in medullary slices revealed that Na_2_S has predominantly depolarizing effects on neurons in superficial laminae of the STN, but in some neurons hyperpolarizing effects occurred. Furthermore, in a previous study it was hypothesized that neuronal NOS and inducible NOS (but not endothelial NOS) were irreversibly inhibited by H_2_S/NO at modest concentrations of H_2_S in a reaction that may allow feedback inhibition of NO production under conditions of excessive NO/H_2_S formation (39).

We cannot definitely exclude that the experimental test setup has influenced the results, since in the majority of experiments i.v. administration was performed after topical application of Na_2_S onto the dura mater and/or the medulla oblongata. However, there was not any significant correlation between the pretreatment and an inhibiting H_2_S effect. We assume that the time intervals between local and systemic application of Na_2_S were long enough to exclude such an interaction.

The lacking change in mechanically evoked activity may indicate an exclusively central effect of Na_2_S/H_2_S on the neurons we have recorded from. Another possibility is that the predominantly activating central effect of H_2_S upon systemic administration of Na_2_S was compensated by the predominantly inhibitory effect on primary meningeal afferents as discussed below.

#### Dural Administration of Na_2_S

After topical application of Na_2_S onto the dura mater, the neuronal activity unexpectedly decreased in the majority (70%) of units within the next 20 min. Analyzing the activity at time intervals of seconds, it turned out that there was a very short-lasting activation (maximally 12 s after application) in the majority of units. This fits to previous recordings from meningeal afferents *in vitro*, where a short-lasting activation under superfusion with the HNO donor Angeli’s salt was followed by a long-lasting inhibition of primary afferent activity (unpublished data of our group). However, this short activation of primary afferents is probably not responsible for the delayed activation seen in the majority of spinal trigeminal units after systemic administration of Na_2_S, because there was no correlation between these effects. In recent recordings in the abovementioned *in vitro* set meningeal primary afferents showed increased thresholds when they were activated by TRPA1 receptor agonists like acrolein or mustard oil (40). Thus, activation of TRPA1 receptors may be the reason why the stimulating effect of H_2_S can eventually decrease the activity of spinal trigeminal neurons. We know from previous studies that the activity of these neurons is partly dependent on an ongoing afferent input, since local anesthesia of trigeminal afferents leads to a significant decrease in activity in these neurons (41).

#### Medullary Administration of Na_2_S

The neuronal response to Na_2_S, administered onto the medulla oblongata, did not lead to a significant effect in the whole sample of recorded units, though in about half of the units the activity increased. This may imply that the neuronal response to H_2_S in the medulla is regulated by further—so far unknown—factors, but it seems more likely that Na_2_S did not reach the cellular environment of the STN at an adequate concentration. Since cerebrospinal fluid (CSF) is produced constantly and diffuses out of the medulla into the cerebrospinal room, Na_2_S is diluted and may chemically react with substances in the CSF before it can diffuse inside the medulla.

### Neuronal Interaction between Na_2_S and DEA-NONOate

The stimulating effect of DEA-NONOate was inhibited by blocking H_2_S synthesis with oxamic acid in 75% of the units and, *vice versa*, blocking NO synthesis by l-NAME reduced the neuronal activity after Na_2_S administration compared with the activity after Na_2_S alone. This indicates a cooperative effect of NO and H_2_S, which does obviously control the activity of most but not all spinal trigeminal neurons. Olcegepant following l-NAME did not lead to a further decrease in neuronal activity, suggesting that the signaling pathway of NO/H_2_S is upstream of central CGRP release in the STN. CGRP has previously been demonstrated to be involved in the nociceptive transmission of signals to second order neurons (42, 43).

#### Systemic Administration of DEA-NONOate

The increased neuronal activity caused by DEA-NONOate is in line with the previously described stimulating effect of “NO donors,” glycerol trinitrate (GNT, nitroglycerin), and sodium nitroprusside, on trigeminal neurons (44, 45) and is on principle consistent with the effect of these substances in triggering headache attacks in patients suffering from migraine (46). However, the stimulating effect of DEA-NONOate appears to be transient, whereas GTN caused a biphasic activation of spinal trigeminal neurons with an acute and a delayed increase in activity starting roughly 1 h after infusion after (44).

The delayed effect of NO on central trigeminal activity may be associated with the expression or availability of CGRP and NO synthase in the trigeminal ganglion or ophthalmic region (47). Thus, NO may have different effects with differing time-course, i.e., an acute effect already occurring during infusion followed by a subacute effect of increased neuronal activity within the next 20 min and a late effect appearing as upregulation of proteins like CGRP, CGRP receptors, and NO synthase some hours after infusion of NO donors (47, 48). Similar changes in the human trigeminal system may eventually trigger headache attacks in migraineurs several hours after i.v. infusion of GTN (49).

#### Systemic Administration of Na_2_S Combined with DEA-NONOate

Diethylamine-NONOate applied 20 min after Na_2_S infusion did not lead to a significant increase in neuronal activity. Conversely, the activity after Na_2_S infusion following 20 min after DEA-NONOate increased rapidly, before it decreased again.

As discussed above, endogenous NO and H_2_S are known to form HNO, which is an effective TRPA1 receptor agonist. HNO stimulates TRPA1 expressing peptidergic sensory neurons to release CGRP and other neuropeptides. Recent experiments in our group have shown that combined stimulation of trigeminal tissues with Na_2_S and NO donors cause CGRP release on all levels of the trigeminal system acting synergistically compared to stimulation with either these substances alone (28). In these preparations, H_2_S appeared to be the rate-limiting factor for HNO formation. This synergistic action of H_2_S and NO was examined in an *in vivo* situation in the present experiments.

Looking closer to the time-course of activation after systemic Na_2_S infusion, the neuronal activity increased particularly during the second 10-min period after Na_2_S administration, whereas after Na_2_S following DEA-NONOate, it increased already during the first 10-min period in the whole sample of units. This may indicate a synergistic action of H_2_S and NO leading to an accelerated activation and supports the hypothesis of an interaction between the two substances with subsequent activation of neurons, most likely by HNO formation. Conversely, DEA-NONOate administered after Na_2_S did not increase the effect of Na_2_S. This difference may be due to the rate-limiting character of Na_2_S, i.e., in the presence of Na_2_S there is sufficient production of NO but not *vice versa*. Thus, infusion of DEA-NONOate after Na_2_S did not lead to a further increase in activity, since the reaction partner H_2_S was missing. In contrast, additional Na_2_S could increase the NO effect and accelerate its stimulating action on trigeminal neurons.

The individual experiments varied again into two directions with stimulated (two-third) and inhibited units (one-third). This is not surprising in the light of the discussion about the Na_2_S effects above.

### Inhibition of Endogenous H_2_S and NO Synthesis

To further analyze the cooperative effect of endogenous NO and H_2_S, we inhibited the enzymes responsible for the synthesis of the reaction partners. We used oxamic acid as an inhibitor of the H_2_S generating CBS (50) and examined the neuronal activity stimulated by DEA-NONOate. Conversely, in another group of experiments, l-NAME was used to explore if the suppression of endogenous NO synthesis has an effect on the Na_2_S-stimulated neuronal activity.

#### Systemic Administration of Oxamic Acid following DEA-NONOate

Infusion of oxamic acid inhibited the majority of units activated by administration of DEA-NONOate in the present study. This provides further evidence for the interaction of NO and H_2_S, which has previously been recognized in other experiments of our group, where the meningeal blood flow increase evoked by systemic application of DEA-NONOate was reduced after topically applied oxamic acid (21). On the other hand, the result may indicate that endogenous H_2_S can also be formed non-enzymatically, even if this may play a minor role [reviewed in Ref. (51)]. Another possibility is that not all neurons are controlled or are even inhibited by the HNO pathway, as discussed above.

#### l-NAME following Systemic Administration of Na_2_S

An inhibitory effect on the ongoing spinal trigeminal activity by the unspecific NOS inhibitor l-NAME was presented previously (29). We applied l-NAME 20 min after i.v. infusion of Na_2_S to further probe the assumed interaction between H_2_S and NO in forming HNO. l-NAME caused a decrease in spontaneous neuronal activity compared to the activity after Na_2_S from minute 6 after the administration; around 20 min after l-NAME the activity fell even below baseline activity. This supports our notion that the activating effect of H_2_S on the majority of spinal trigeminal neurons depends on the presence of NO.

Calcitonin gene-related peptide has been identified as a predominantly pro-nociceptive neuromodulator by iontophoretic application of CGRP and CGRP receptor antagonists close to the recorded neurons in the trigemino-cervical brainstem complex (42). Following l-NAME administration we eventually applied a CGRP receptor inhibitor, Olcegepant (BIBN4096BS), with proven therapeutic efficiency in acute migraine headache (52). This did not lead to a further decrease in neuronal activity, fitting the hypothesis that CGRP is released by TRPA1 activation upon HNO formed by NO and H_2_S. When NO production was interrupted by l-NAME, HNO formation and hence CGRP release was presumably reduced in the STN with the consequence of decreased neuronal activity, as demonstrated earlier (29).

## Conclusion

Spinal trigeminal neurons may be activated or inhibited by H_2_S–NO–CGRP signaling. Although direct actions of H_2_S and NO cannot be excluded, we collected strong evidence for a cooperative effect of H_2_S and NO forming HNO, which upon TRPA1 activation modulates CGRP release and the activity of these neurons. Endogenous NO production may contribute more to the maintenance of spinal trigeminal activity than endogenous H_2_S production, while the latter may be the rate-limiting factor. Our *in vivo* experiments point to divergent responses to H_2_S of neurons in the trigeminal system. We suggest similar divergent effects of this neuromodulator when it is endogenously produced in the human body. Depending on its blood concentration and the interaction with other modulators like NO, it may be an important factor for the generation of migraine pain and tension-type headaches. Progress in understanding the role of H_2_S in trigeminal nociception may support further pharmacological studies of the H_2_S–NO–CGRP system aiming at new options in the therapy of primary headaches.

## Ethics Statement

The experiments were approved by the District Government of Unterfranken, Bavaria.

## Author Contributions

CT performed the experiments, analyzed the data, and wrote the manuscript. RC instructed the experimental work and data analysis and contributed to the manuscript. KM designed and supervised the experiments and wrote the manuscript.

## Conflict of Interest Statement

The authors declare that the research was conducted in the absence of any commercial or financial relationships that could be construed as a potential conflict of interest.

## Abbreviations

CBS, cystathionine β-synthase; CGRP, calcitonin gene-related peptide; CO, carbon monoxide; CSE, cystathionine γ-lyase; DEA-NONOate, diethylamine-NONOate; H_2_S, hydrogen sulfide; HNO, nitroxyl; l-NAME, nitro-l-arginine methyl ester hydrochloride; Na_2_S, sodium sulfide; NO, nitric oxide; NOS, nitric oxide synthase; SIF, synthetic interstitial fluid; STN, spinal trigeminal nucleus; TRPA1, transient receptor potential channel of the ankyrin type 1; TRPV1, transient receptor potential channels of the vanilloid type 1.
